# Wavelet-based background and noise subtraction for fluorescence microscopy images

**DOI:** 10.1364/BOE.413181

**Published:** 2021-01-22

**Authors:** Manuel Hüpfel, Andrei Yu. Kobitski, Weichun Zhang, G. Ulrich Nienhaus

**Affiliations:** 1 Institute of Applied Physics, Karlsruhe Institute of Technology (KIT), Wolfgang-Gaede-Str. 1, 76131 Karlsruhe, Germany; 2 Institute of Nanotechnology, Karlsruhe Institute of Technology (KIT), Hermann-von-Helmholtz-Platz 1, 76344 Eggenstein-Leopoldshafen, Germany; 3 Institute of Biological and Chemical Systems, Karlsruhe Institute of Technology (KIT), Hermann-von-Helmholtz-Platz 1, 76344 Eggenstein-Leopoldshafen, Germany; 4 Department of Physics, University of Illinois at Urbana-Champaign, 1110 W. Green Street, Urbana, IL 61801, USA

## Abstract

Fluorescence microscopy images are inevitably contaminated by background intensity contributions. Fluorescence from out-of-focus planes and scattered light are important sources of slowly varying, low spatial frequency background, whereas background varying from pixel to pixel (high frequency noise) is introduced by the detection system. Here we present a powerful, easy-to-use software, wavelet-based background and noise subtraction (WBNS), which effectively removes both of these components. To assess its performance, we apply WBNS to synthetic images and compare the results quantitatively with the ground truth and with images processed by other background removal algorithms. We further evaluate WBNS on real images taken with a light-sheet microscope and a super-resolution stimulated emission depletion microscope. For both cases, we compare the WBNS algorithm with hardware-based background removal techniques and present a quantitative assessment of the results. WBNS shows an excellent performance in all these applications and significantly enhances the visual appearance of fluorescence images. Moreover, it may serve as a pre-processing step for further quantitative analysis.

## 1. Introduction

**Fig. 1.**
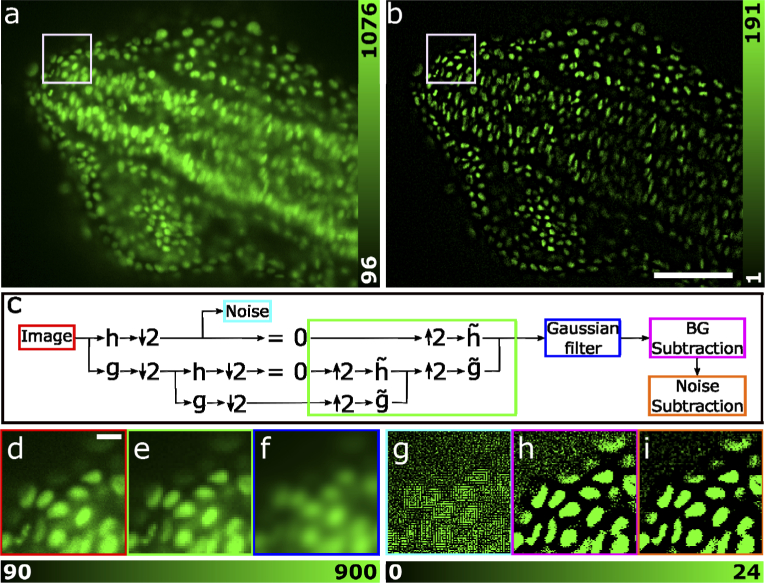
The WBNS software and its application to fluorescence images. (a) DSLM image from a 3D image stack showing cell nuclei of a zebrafish embryo (head section) labelled with GFP. Data are taken from Ref. [6]. (b) The corresponding image after processing with WBNS. Scale bar, 100 µm. (c) Flowchart of the WBNS algorithm. First, the input image (red box) is decomposed by consecutively applying two wavelets acting as high-pass (h) and low-pass (g) filters, followed by subsampling by a factor of two (↓2). The details of the first level contain high-frequency noise (cyan box) on characteristic length scales smaller than the PSF. This decomposition is repeated multiple times, here we show only two levels for brevity. Higher level approximations contain the low-frequency background. We set the details to zero and reconstruct an image by upsampling (↑2) and applying the inverse wavelet transform ($\tilde{\text{g}},\;\tilde{\text{h}}$) at all levels to obtain the background estimate (green box). A Gaussian filter (blue box) smooths discontinuities. Finally, the background (magenta) and the noise (orange) are subtracted from the image. (d)-(i) Close-ups of the image (white boxes in panels a and b) during different stages of the algorithm. The colors of the image frames agree with those of the corresponding boxes in panel (c). (d) Original image (scale bar, 8 µm), (e) unfiltered and (f) Gaussian filtered background estimate, (g) extracted noise image, (h) image after background subtraction and (i) image after background and noise subtraction. Panels (g), (h) and (i) are shown with enhanced contrast to visualize the effects on the noise. The pixel intensities are encoded as indicated by the color bars below the images.

Over the past decades, fluorescence microscopy has developed into a key enabling experimental technique in life sciences research. Especially the advent of super-resolution microscopy techniques in recent years has stirred enormous excitement, as these powerful new methods offer entirely new opportunities to explore biological processes at the subcellular level [1–3]. A wide variety of imaging modalities have become available that markedly differ in regard to their spatial and temporal resolution, signal-to-background ratio and sample health due to light exposure. Thus, the particular technique has to be wisely chosen to best fulfil the demands of an imaging experiment aimed at solving the biological question at hand. Ideally, fluorescence microscopy images depict structures of biological samples as (mathematical) convolutions with the point spread function (PSF) of the microscope, which introduces blur due to the limited spatial resolution of the microscope (caused by the diffraction of light in classical microscopy). Real images are, in addition, contaminated by low spatial frequency background intensity, especially due to out-of-focus fluorescence and scattered light. Moreover, the detection process introduces high-frequency noise that further deteriorates the image quality (Fig. 1(a)). Various approaches have been devised to suppress these adverse effects, either as hardware-based modifications to an existing microscope design or software solutions for post-processing of images. Here we have developed a powerful, wavelet-based background and noise subtraction (WBNS) algorithm that removes background as well as noise from the image (Fig. 1(b)). To evaluate its performance, we have processed synthetic ground-truth images with WBNS and compared the results quantitatively with the ground truth and images processed by other background removal algorithms. We have further applied WBNS to real fluorescence images. To this end, we have selected, on the one hand, a widefield technique with camera detection, digital scanned light sheet microscopy (DSLM) [4] and, on the other hand, a raster scanning confocal technique capable of super-resolution, namely stimulated emission depletion (STED) microscopy [5]. For both modalities, hardware-based background removal techniques are also available, so that we can compare the efficacy of the hardware and software solutions in a quantitative fashion.

Here we demonstrate that the WBNS program is a powerful tool that can be applied to all sorts of images, regardless of the particular imaging modality chosen. It is versatile and easy to use as it requires as input only a two-dimensional (2D) image or three-dimensional (3D) image stack plus a single additional parameter, *R*, the full width at half-maximum (FWHM) of the PSF, which specifies the optical resolution.

## 2. Wavelet-based image analysis algorithm

WBNS uses the discrete wavelet transform to decompose the image into high- and low-frequency components at multiple, logarithmically spaced resolution levels. Brief, insightful introductions to this algorithm, which can be implemented elegantly as a digital filter bank, can be found in Refs. [7–9]. We employ the small Haar wavelet to transform the information contained in the image into high-frequency components, the so-called “detail coefficients”, and low-frequency components, the “approximation coefficients”, by consecutively applying the wavelets as high-pass and low-pass filters, respectively. Thereby, the pixel number is implicitly reduced by a factor of two in both dimensions. The low-pass filtered image serves as input for the next level of analysis. This process is repeated for *m* ≤ log_2_(*n*) levels, *n* being the image size in pixels. A user-specified parameter, *R*, controls the number of decomposition levels, *m* = ⌈log_2_(*R*)⌉, and thus defines the spatial frequency cut-off between image information and background. The standard choice for *R* is the FWHM of the PSF (in units of pixels) as a measure of image resolution. However, *R* can be increased if the image content is broadly dispersed in frequency to avoid assignment of lower frequency information to background. As a result, background removal becomes less effective (see below). An image containing only low-frequency background is reconstructed by setting the detail coefficients in all levels of the decomposed images to zero and applying the inverse wavelet transform to all levels. The resulting background image is low-pass filtered and subtracted from the original one to yield the background-cleared image. The detail coefficients of the first level represent high-frequency noise produced by the detection system (more levels may be necessary for low-resolution images). This component is extracted by setting the approximation coefficients to one so that an image containing only high-frequency noise is reconstructed from the first-level details, which is subsequently subtracted from the original image for noise removal. A diagram of the algorithm is depicted in Fig. 1(c) (for two levels of wavelet decomposition). To illustrate its action on the images, close-ups calculated during different stages of processing are shown in Fig. 1(d)-(i). Further details of the WBNS algorithm are included in the flow chart in Fig. S1 and in the associated caption. In the remainder of the paper, we refer to the algorithm with switched-off noise suppression as wavelet-based background subtraction (WBS).

Why did we use the simple Haar wavelet transform for WBNS rather than more sophisticated multiresolution analysis methods that have been introduced for image processing, which are typically based on or related to the wavelet transform? For example, the curvelet and the contourlet transforms have been developed to capture smooth curves and edges and, therefore, are excellent for processing densely pixeled, oversampled images such as photographs [10,11]. In biological fluorescence microscopy, however, oversampling is usually avoided as it decreases the signal-to-noise ratio. In this case, the Haar wavelet transform is advantageous because it offers the smallest support (two pixels), allowing us to separate the characteristic length scales of noise (one or two pixels), signal (in fluorescence microscopy images typically between three and five pixels, as a trade-off between image resolution and signal-to-noise ratio) and background (more than five pixels). Moreover, in comparison to other approaches, the Haar wavelet transform is conceptually simple, computationally cheap and memory efficient.

## 3. Methods

### 3.1. Sample preparation

For DSLM imaging, a stock suspension of red-fluorescent carboxylated polystyrene beads (excitation/emission wavelengths: 580/605 nm) with a nominal diameter of 100 nm (F8801, FluoSpheres, Invitrogen, Eugene, OR) was diluted 10^5^-fold in water, sonicated for 10 min to reduce the number of aggregates and then further diluted in an aqueous solution containing 1.5% (by mass) low melting point agarose (type VII, A6560, Sigma Aldrich, St. Louis, MO) to a concentration at which individual spots were clearly resolved in 3D DSLM images. The liquid agarose solution was again sonicated for 10 min and then filled into a fluorinated ethylene propylene (FEP) tube (Thomafluid HighTech Tubing, outer/inner diameters 1.5/1.1 mm, Reichelt Chemietechnik GmbH, Heidelberg, Germany) with a syringe. After cooling and polymerization, part of the gel (ca. 2 mm) was gently extruded from one end of the tube for imaging without the surrounding FEP tube to exclude optical aberrations introduced by the FEP tube. The tube was attached to a stainless steel rod, mounted on the rotor stage and dipped into the sample chamber from above.

For confocal and stimulated emission double depletion (STEDD) imaging, an 8-well chambered cover glass (thickness: 0.16–0.19 mm) with non-removable wells (155409, Nunc Lab-Tek II, Thermo Scientific, Waltham, MA) was incubated with 0.1 mg mL^-1^ poly-L-lysine (PLL, P6282, Sigma-Aldrich) dissolved in water for 20 min. PLL is a positively charged polymer that tightly attaches to the glass surface so as to form an adhesive layer suitable for immobilizing negatively charged (carboxylated) fluorescent beads. A stock solution of dark red polystyrene beads (excitation/emission wavelengths: 660/680 nm) with a nominal diameter of 40 nm (F8789, FluoSpheres, Invitrogen) was diluted by a factor of 50,000 with phosphate-buffered saline (PBS) (14040091, Thermo Scientific). The suspension was sonicated for 10 min, added to the PLL-treated sample chamber and allowed to react for 20 min. The chamber was washed 3× with PBS to remove unbound fluorescent beads. Finally, the sample chamber was filled with PBS and stored at 4°C until use.

### 3.2 DSLM image acquisition

Our current, home-built DSLM is a significantly upgraded version of the device described in Ref. [6], featuring Bessel beam illumination [12] and confocal slit detection [13] (DSLM-CS, Fig. S2). We recorded DSLM image stacks of 100 slices with a mutual spacing of 500 nm and a frame rate of 10 s^-1^ of 100 nm fluorescent beads immobilized in agarose gel. Each slice consists of 2048 × 2048 pixels covering a field of view of 176 µm × 176 µm. An image measured beforehand with all excitation lasers switched off was subtracted from each slice to remove dark camera background.

### 3.3 Confocal and STEDD image acquisition

We collected confocal and STEDD images (604 × 604 pixels, pixel dwell times 30 and 100 µs, respectively, pixel size 10 nm) of 40 nm dark red beads immobilized on glass surfaces with our home-built STED microscope [14]. Fluorescence was excited by a picosecond laser delivering 640-nm pulses at 80 MHz (16 µW). For STEDD imaging, the excitation pulse was followed by two 736-nm pulses from a Ti:Sa laser in succession, the first one (STED1) with a power of 39 mW was shaped to a “donut” intensity distribution by a vortex phase mask; the second one (STED2), with a power of 3.9 mW and a time delay of 3 ns with respect to STED1, was simply generated by focusing the Gaussian laser beam. The STEDD method enables synchronized measurement of a STED image (in the interval between the STED1 and STED2 pulses) and a background image (after the STED2 pulse, which erases the high-frequency signal and leaves low-frequency background) [14]. To obtain the STEDD image, the background image is subsequently subtracted from the STED image with an appropriate weight factor (here, γ = 2) determined as described in Ref. [15]. To reduce noise, the heavily oversampled background image was processed with a Gaussian filter of standard deviation σ = 15 pixels before subtraction. For further details of the STEDD method, we refer to Ref. [14].

### 3.4 Software implementation

We implemented the WBNS algorithm in Python 3.7, using the open source “PyWavelets” software [16]. Furthermore, we developed an easy-to-use ImageJ macro and we also implemented the algorithm as a MATLAB R2019b (The MathWorks, Natick, MA) function. These implementations are freely available via Github https://github.com/NienhausLabKIT/HuepfelM. Software implementation guides for Python and ImageJ are included as Supplementary Notes 1 and 2.

## 4. Performance assessment of WBNS using synthetic images

**Fig. 2.**
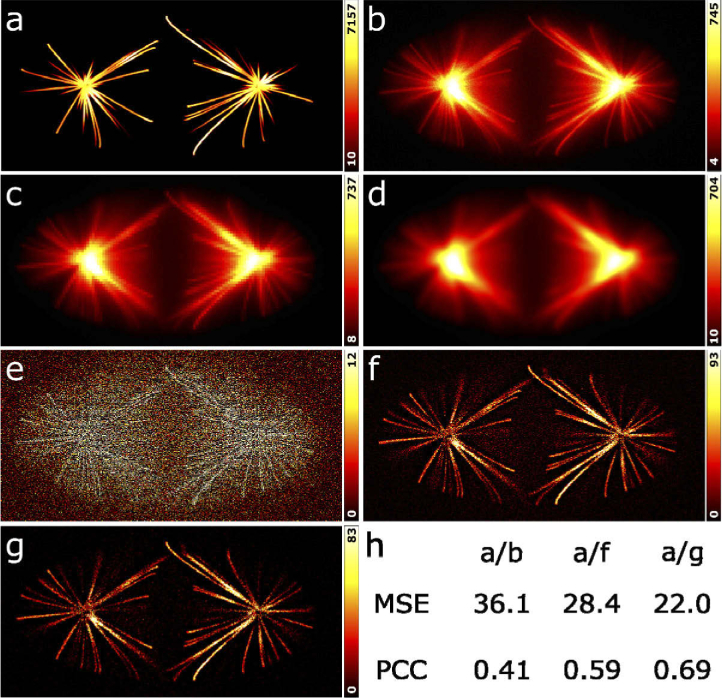
WBS/WBNS application to simulated data. (a) Single slice of a synthetic, 3D ground-truth image stack (512 × 256 × 128 pixels). (b) Single slice of a “pseudo” microscopy image stack, calculated from the synthetic image by convolution with a synthetic PSF (lateral FWHM = 3 pixels) and addition of Poissonian (average value *λ* = 1) and Gaussian noise (mean *µ* = 0 and standard deviation *σ* = 1). (c) Background estimate from wavelet decomposition (*R* = 4 pixels). Close inspection shows discontinuities between neighboring regions arising from the use of the (discontinuous) Haar wavelet. (d) Background estimate after Gaussian filtering with *σ* = 2*^m^*, where *m* is the number of decomposition levels, so that transitions are smoothened across the characteristic scale of the background. (e) High-frequency noise extracted from the details of the first level of wavelet decomposition. Outlier pixels were clipped to *µ *+ 2*σ* to reduce artefacts. Processed images with (f) subtraction of background only (WBS) and (g) noise subtraction in addition (WBNS), showing more prominent structural details and markedly reduced noise. (h) Mean square error (MSE) and Pearson correlation coefficient (PCC) [18], calculated between the ground-truth image (panel (a)) and the “pseudo” microscope image (panel (b)), the background corrected image (panel (f)) and the background and noise corrected image (panel (g)). All images have been normalized to the same mean intensity.

To assess the performance of WBNS, we started with a synthetic, 3D ground-truth image stack (512 × 256 × 128 pixels) [17]. A representative slice from the middle of the stack is shown in Fig. 2(a). We turned this ground-truth 3D image into a “pseudo” microscopy image by first convolving it with a realistic 3D PSF using the software DeconvolutionLab2 [17]. The shape of the PSF is depicted in Fig. S3 along with selected cross sections, illustrating the broadening and background generating effect of the convolution. In addition, we have added Poissonian and Gaussian noise (Fig. 2(b), Visualization 1) [17]. Setting the parameter *R* to 4 pixels, as is appropriate for the chosen PSF, we processed all images of the stack by wavelet decomposition in three levels (as depicted in Fig. S1). Then, we set all detail coefficients to zero and resynthesized the images to obtain a background estimate (Fig. 2(c)). The use of the discontinuous Haar wavelet produces discontinuities in the background intensity; therefore, we included Gaussian filtering of background images into the WBNS program, with standard deviation *σ* = 2*^m^*, where *m* is the number of decomposition levels, so that transitions are smoothened across the characteristic scale of the low-frequency background (Fig. 2(d)). A high-frequency noise image was calculated by resynthesizing the first-level wavelet-decomposed images with all approximation coefficients set to 1 (Fig. 2(e)). We note that oversampled images, which have too many pixels in comparison to the resolution, may need two (or even more) levels of wavelet decomposition for this analysis. In the noise images, negative pixel values were set to zero, and outliers, i.e., pixels with abnormally high values, were limited to the mean plus twice the standard deviation (*µ *+ 2*σ*) to reduce artefacts. The original image (Fig. 2(b)) with the background image subtracted is shown in Fig. 2(f). Additional noise subtraction yields the image shown in Fig. 2(g) (Visualization 1), in which the structural details are prominent and the noise is markedly reduced. For a quantitative assessment of WBNS image processing, we calculated the mean square error (MSE) and the Pearson correlation coefficient (PCC) [18] between the ground-truth image (Fig. 2(a)) and the “pseudo” microscopy image (Fig. 2(b)), the background corrected image (Fig. 2(f)) and the background and noise corrected image (Fig. 2(g)). To this end, all images were normalized to the same mean intensity. Both quantitative measures indicate that the background corrected image has a markedly higher similarity to the ground truth image than the “pseudo” microscopy image (Fig. 2(b)), and noise clearing further increases the similarity (Fig. 2(h)).

**Fig. 3.**
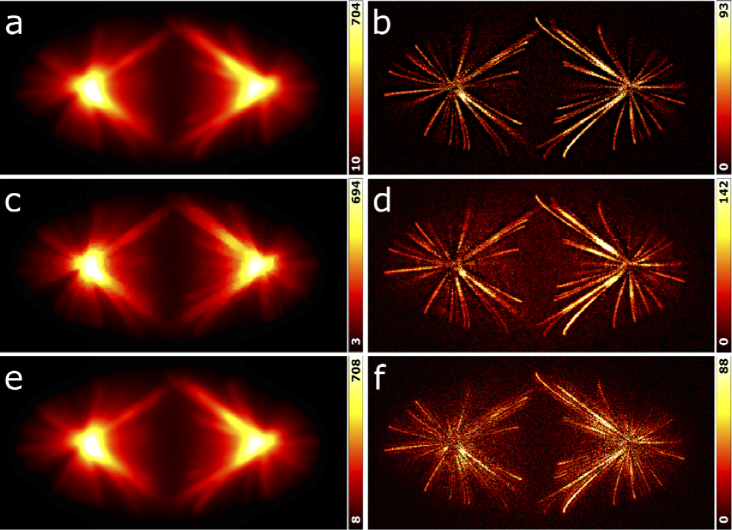
Comparison of WBS with the RBA and DoG background removal algorithms based on the synthetic image in Fig. 2(b). (a) Background estimate of WBS; *R* = 4 pixels. (b) Image after background removal with WBS. (c) Background estimate of RBA using an ImageJ plugin (https://imagej.nih.gov/ij/plugins/rolling-ball.html) and a ball radius of 4 pixels. (d) Image after background removal with the RBA. (e) Background estimate of the DoG method, using a standard deviation of 4 pixels. (f) Image after background removal with the DoG method.

We next asked how our wavelet-based method compares with other low-frequency background clearing algorithms. We selected the rolling ball algorithm (RBA) [19] and the difference of Gaussians method (DoG) [20] for this test. In the RBA, a sphere with a specified radius is moved underneath and in permanent contact with the intensity landscape corresponding to the image. As the sphere cannot intrude into narrow features (signal), the algorithm effectively acts as a low-pass filter and returns a background image. In the DoG algorithm, a background image is calculated by blurring the original image with a Gaussian filter, which is then subtracted from the original image. Figure 3 shows, for the three algorithms, the background images on the left and the background-subtracted images on the right. Of course, we used WBS (without high-frequency noise suppression) here for a fair comparison. Visual inspection of the cleared images shows that all three methods effectively remove background. For a quantitative comparison, we calculated the similarity measures MSE and PCC of the background-subtracted images with respect to the ground truth (GT) image for the entire 3D image stack. The data in Table 1 show that WBS performs best in this comparison, as judged from the lowest MSE and highest PCC values of the three methods. Additional noise subtraction using WBNS leads to an even closer similarity to the ground truth.

**Table 1.** Comparison of the ground truth (GT) image with background-cleared images calculated with the WBNS, RBA and DoG methods by using MSE and PCC as quality measures.

	GT / RBA	GT / DoG	GT / WBS	GT / WBNS
MSE	34.7	34.3	29.6	22.0
PCC	0.50	0.48	0.57	0.69

## 5. Application of WBNS to DSLM images

**Fig. 4.**
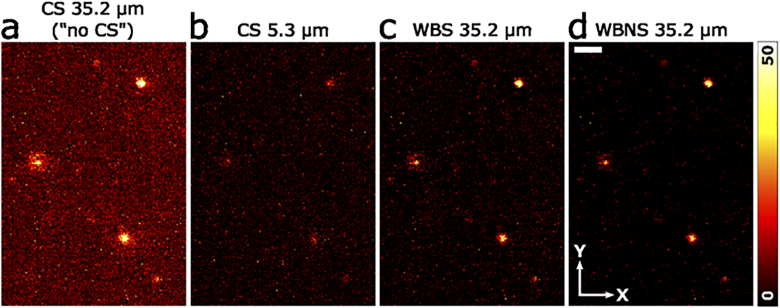
Comparison between WBS/WBNS and DSLM-CS. (a)-(d) Close-up views (153 × 219 pixels) of an exemplary slice from a 3D DSLM image of 100-nm fluorescent polystyrene beads immobilized in agarose gel, acquired using (a) a wide detection slit (35.2 µm, *w*_slit_ >> *w*_beam_), so that there is high background due to the lacking rejection of the emission from outside the focal plane. (b) With a narrow slit (5.3 µm, *w*_slit_ ≈ *w*_beam_), background is markedly suppressed due to CS detection. Image in panel (a) processed with (c) WBS and (d) WBNS (*R* = 5.5 pixels, noise subtraction based on the first detail level). Scale bar, 2 µm.

For a performance assessment of WBNS in widefield imaging, we acquired 3D image stacks covering a volume 176 × 176 × 50 µm^3^ of 100-nm fluorescent beads in agarose gel on our home-built DSLM setup (Fig. S2). It employs a Bessel beam [12] that is laterally scanned to form a light sheet for illumination; the fluorescence is detected by a camera capable of confocal slit (CS) detection, a well-established hardware-based modality for background reduction [13]. For each 3D image, 100 camera frames (2048 × 2048 pixels) were taken along the *z*-direction with a mutual spacing of 500 nm and a frame rate of 10 s^-1^. A dark (laser off) image was subtracted from each slice to remove dark camera background. A first image stack was measured with a wide detection slit (35.2 µm, *w*_slit_ >> *w*_beam_) so that there was no confocal effect, i.e., axial sectioning, implying that background emission from outside the focal plane reaches the camera. The second stack was taken with a narrow slit (5.3 µm, *w*_slit_ ≈ *w*_beam_) causing effective suppression of background due to CS detection, as is evident from comparing close-ups from a single image slice in Fig. 4(a) and (b). The image stack taken with a wide slit was processed with WBS and WBNS (Fig. 4(c),(d)); the effective background and noise clearing capability of the software is obvious from comparison with Fig. 4(a). For a quantitative analysis of hardware- and software-based background suppression, we determined the apparent bead sizes, which tend to increase in the presence of background and noise, from the 3D image stacks. To this end, we identified and preselected local intensity maxima by their prominence, size and brightness so as to avoid inclusion of aggregates. For each bead image, we quantified its extensions (FWHM) along all three axes (through the center position of each bead) to be about 0.5 µm in the image plane (*x*, *y*) and 2 µm perpendicular to the image plane (*z*). All FWHM values were compiled in cumulative histograms and fitted by the cumulative normal distribution function to obtain mean values and standard deviations over ensembles of 63–77 beads (Table 2). The data clearly show that the axial sectioning of DSLM-CS reduces the apparent extensions of the beads markedly, especially along the *z*-direction (>12%). Comparison of raw DSLM-CS images with those processed by WBS reveals that software processing resulted in bead sizes that were slightly smaller than those from DSLM-CS, suggesting that WBS is even more effective for background reduction than DSLM-CS. Finally, WBNS can also be applied to DSLM-CS images such as Fig. 4(b) and leads to significant noise reduction (Fig. S4).

**Table 2.** Apparent sizes of 100-nm fluorescent latex beads in an agarose gel from DSLM image stacks measured with two confocal slit (CS) widths, without and with processing by WBS/WBNS.

	FWHM *x* / nm	FWHM *y* / nm	FWHM *z* / nm	# beads
CS 35.2 µm (“no CS”)	530 ± 80	520 ± 70	2450 ± 380	77
CS 5.3 µm	480 ± 110	500 ± 120	2150 ± 560	66
WBS 35.2 µm	440 ± 80	460 ± 70	2090 ± 330	63
WBNS 35.2 µm	440 ± 80	460 ± 70	2040 ± 340	63

## 6. Application of WBNS to STED images

**Fig. 5.**
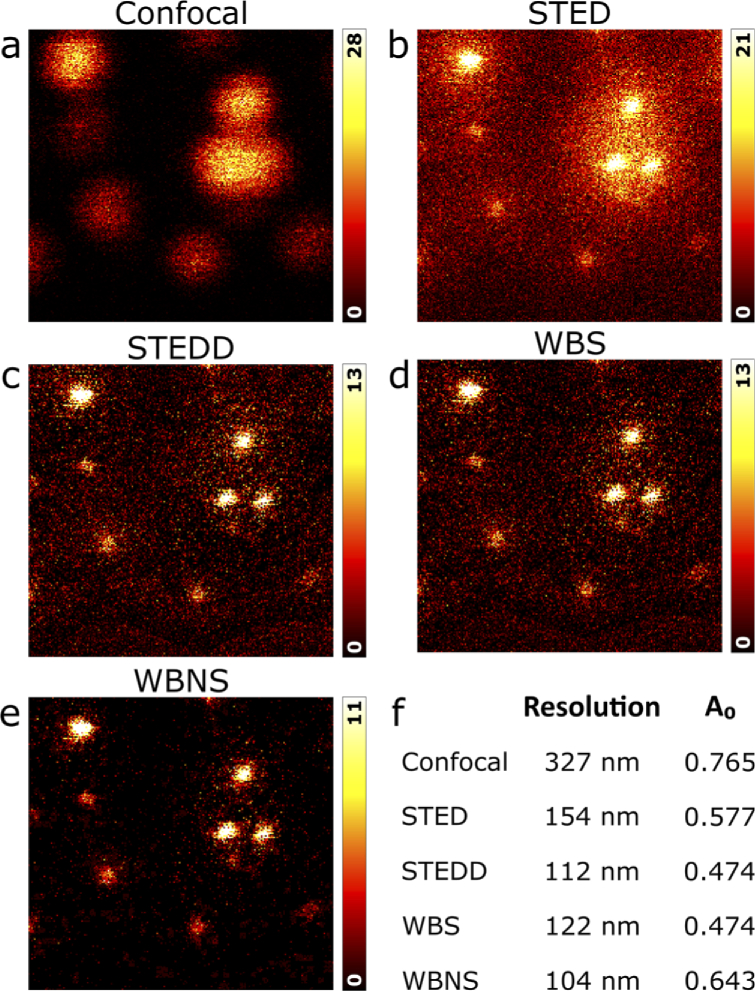
Comparison between WBS/WBNS and STEDD. (a)-(e) Close-ups (219 × 210 pixels) of the same region of a 2D image (604 × 604 pixels, pixel size 10 nm) of surface-immobilized 40-nm fluorescent polystyrene beads, visualized as (a) confocal image, (b) regular STED image and (c) STEDD image. (d) The STED image processed with WBS (*R* = 7 pixels) closely resembles the STEDD image. (e) The STED image processed with WBNS (noise: first detail level) additionally shows reduced high-frequency noise. (f) Image resolution and parameter, *A*_0_ (correlated with the SNR), as obtained by decorrelation analysis using an ImageJ plugin (https://github.com/Ades91/ImDecorr) [22].

To assess the performance of WBNS for background and noise removal from super-resolution STED images, we imaged 40-nm fluorescent polystyrene beads immobilized on glass surfaces. In STED microscopy [5], a tightly focused Gaussian laser beam is raster-scanned across the sample to excite fluorescence. This spot is spatially and temporally overlaid with a red-shifted, high-power beam focused to a “donut” intensity distribution, which selectively deexcites fluorophores in the periphery of the excitation spot by stimulated emission. Thus, the scanning probe is sharpened because only fluorophores within the central region of the spot remain electronically excited after application of the depletion pulse. Apart from out-of-focus and scattered light, STED images always include an additional low-frequency background component that originates from incomplete depletion as well as reexcitation by the powerful depletion beam. We recently introduced stimulated emission double depletion (STEDD) as a hardware-based technique that effectively removes this component [14], which facilitates super-resolution fluorescence correlation spectroscopy experiments on solution samples, and also greatly helps remove background in 3D imaging of densely labeled structures [21]. The significant resolution enhancement between confocal and STED/STEDD modes and the effective background suppression of STEDD are obvious from visual inspection of the images taken on our 40-nm bead samples (Fig. 5(a)-(c)). Processing the STED image with WBS (*R* = 7 pixels, noise: first detail level) resulted in an image (Fig. 5(d)) closely resembling the STEDD image (Fig. 5(c)). High-frequency noise is strongly reduced when applying WBNS to the STED image (Fig. 5(e)). For a quantitative comparison of the images, we determined the image resolution by decorrelation analysis using an ImageJ plugin [22]. In addition, this software also provides a parameter, *A*_0_, which is positively correlated with the signal-to-noise ratio (SNR); however, it is presently not yet clear if *A*_0_ can serve as a SNR metric [22]. The results, presented in Fig. 5(f), reveal that the regular STED image has a more than two-fold higher image resolution over confocal microscopy. STEDD leads to a further improvement to about three-fold because low-frequency components in the power spectrum of the image are suppressed. With respect to STEDD, processing of the STED image with WBS and WBNS yields similar, in fact slightly worse and better resolutions, respectively (Fig. 5(f)). The *A*_0_ values successively decrease in the sequence confocal, STED, and STEDD imaging. This behaviour reflects the overall numbers of photon counting events that make up the image. While the largest number of emitted photons is collected by confocal microscopy, their number decreases for STED imaging due to a large fraction of depletion events, which is the price to pay for the higher resolution, and even more so for STEDD due to the additional background subtraction. Interestingly, we obtain identical *A*_0_ values for STEDD and WBS-processed images, whereas WBNS shows a greater *A*_0_, as expected upon high-frequency noise removal. WBNS results for the confocal image shown in Fig. 5(a) are included in Fig. S5 for completeness. To summarize, we found a comparable performance for background removal with the WBNS software and the hardware solution STEDD on these images. Naturally, WBNS can also be applied to STEDD images such as Fig. 5(c), yielding significant noise reduction (Fig. S6).

## 7. Conclusion

The presence of artificial intensity contributions in fluorescence microscopy images can greatly deteriorate and even entirely obfuscate their information content. As a consequence, considerable efforts have been devoted to background and noise suppression, either by introducing special hardware into the imaging system or by developing software for post-processing of the images. Here we have presented WBNS, a software for background and noise removal from microscopy images, which employs multiscale wavelet decomposition of the experimental image to identify background and noise. Resynthesis of two images containing high-frequency (noise) and low-frequency (background) components allows their subsequent subtraction from the original image. To properly remove noise artifacts, the algorithm uses prior knowledge, i.e., that the microscope itself is an optical low pass filter and cannot transmit spatial frequencies beyond a certain limit, i.e., the diffraction limit in classical microscopy. Thus, intensity components with higher spatial frequencies than allowed by the PSF must be artefacts and originate from the detection system; they are assigned to noise. For low-frequency background, the situation is more ambiguous because there is no clear-cut separation between signal and background based on physical law. However, objects of interest, e.g., biological samples, are often well structured and feature a fairly narrow band of spatial frequencies. By choosing the adjustable parameter *R* to be the image resolution in pixels, blurry intensity from outside the focal plane or light scattering is assigned to low-frequency background. However, if the object of interest presents a wide spectrum of spatial frequencies, the parameter *R* must be increased to avoid removing lower-frequency image components as background. As a consequence, the separation between image and background is shifted to lower spatial frequencies and background can only partially be suppressed. To illustrate this important issue, we have processed images of a developing zebrafish embryo containing both sharp and broad image features with WBNS (Fig. S7). Variation of *R* shows how preserving the low-frequency image information by increasing *R* results in less effective background removal. Notably, the examples shown in Fig. S7 are somewhat special, featuring an enormous bandwidth in their image content. In most applications, however, WBNS will just work well using *R* as the image resolution in pixels. Indeed, in our ongoing biological projects, WBNS has shown great promise, enhancing the visual appearance of a variety of images without deteriorating their content.

In recent years, deep learning-based algorithms have gained considerable popularity for image restoration purposes including background removal [23–26]. These algorithms require training with suitable image data displaying the desired image features, i.e., real microscopy images or synthetic data [24]. The performance of the neural network and thus the accuracy of image restoration crucially depend on the quality and suitability of the training set for the particular image content. WBNS is a general and conceptually simple yet sophisticated filtering algorithm that is applicable to any microscopy image or 3D image stack. Accordingly, the resulting modifications applied to the images are completely traceable, in contrast to deep learning-based algorithms.

To summarize, we have quantitatively compared WBNS to other algorithms and imaging modalities that provide hardware-based background suppression, and we have observed an excellent performance. The software is easy to use, as it only requires a single additional parameter specifying the spatial-frequency separation between image and background. Therefore, we hope that this program will be widely appreciated by the imaging community.

## Supplemental document

See Supplement 1 for supporting content.
