# Supplementary material for: Wavelet-based background and noise subtraction for fluorescence microscopy images
Source: Biomed Opt Express. 2021 Jan 22;12(2):969–80. doi: 10.1364/BOE.413181 (PMC7901331; doi:10.1364/BOE.413181)
Supplement: Supplementary file 2 [file boe-12-2-969-s001.pdf]

Supplemental Document

Biomedical Optics EXPRESS

Wavelet-based background and noise subtraction for fluorescence microscopy images: supplement

MANUEL HÜPFEL,[1] ANDREI YU. KOBITSKI,[1] WEICHUN ZHANG,[1] AND G. ULRICH NIENHAUS[1,2,3,4,*]

[1] *Institute of Applied Physics, Karlsruhe Institute of Technology (KIT), Wolfgang-Gaede-Str. 1, 76131 Karlsruhe, Germany*
[2] *Institute of Nanotechnology, Karlsruhe Institute of Technology (KIT), Hermann-von-Helmholtz-Platz 1, 76344 Eggenstein-Leopoldshafen, Germany*
[3] *Institute of Biological and Chemical Systems, Karlsruhe Institute of Technology (KIT), Hermann-von-Helmholtz-Platz 1, 76344 Eggenstein-Leopoldshafen, Germany*
[4] *Department of Physics, University of Illinois at Urbana-Champaign, 1110 W. Green Street, Urbana, IL 61801, USA*
[*] *uli@uiuc.edu*

This supplement published with The Optical Society on 22 January 2021 by The Authors under the terms of the Creative Commons Attribution 4.0 License in the format provided by the authors and unedited. Further distribution of this work must maintain attribution to the author(s) and the published article's title, journal citation, and DOI.

Supplement DOI: https://doi.org/10.6084/m9.figshare.13582814

Parent Article DOI: https://doi.org/10.1364/BOE.413181

Wavelet-based background and noise subtraction for fluorescence microscopy images: supplemental document

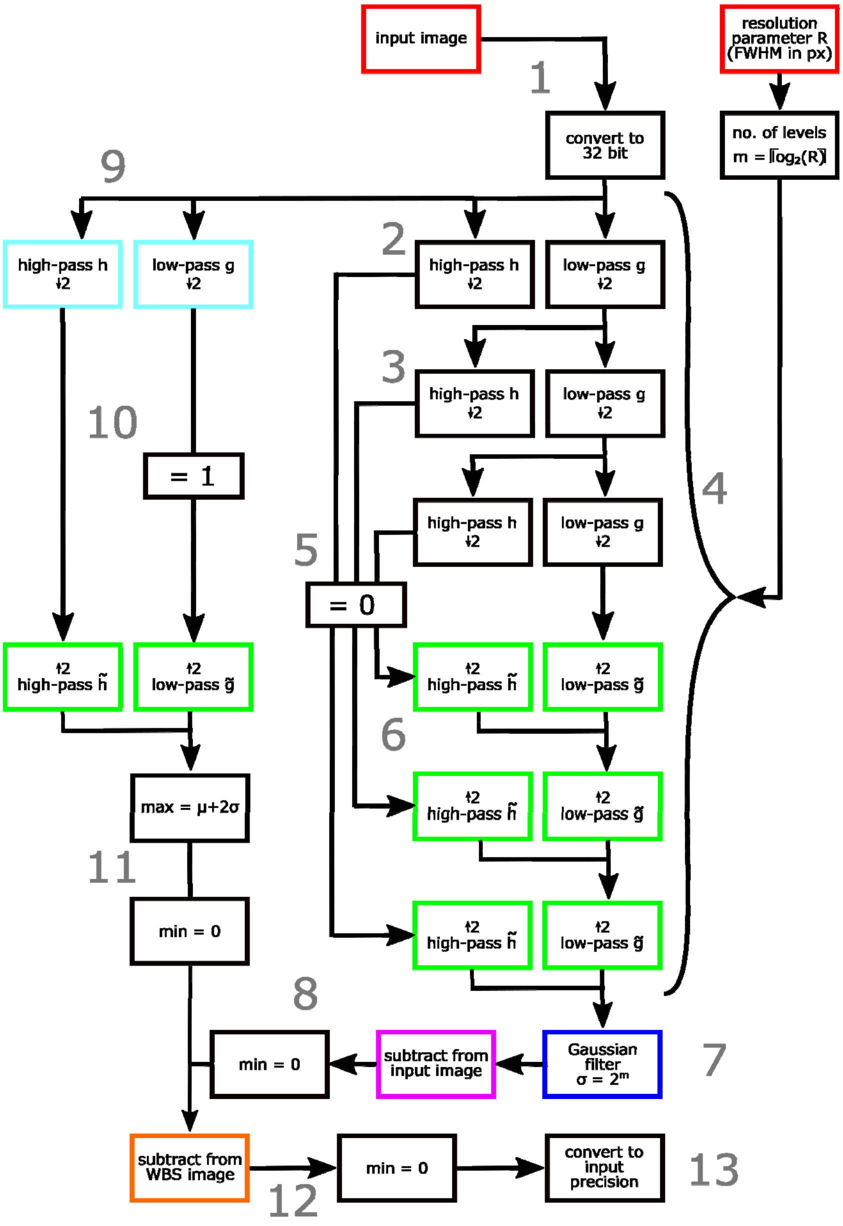

Fig. S1: Flowchart of the WBS/WBNS algorithm. Shown is a detailed schematic of the wavelet-based background subtraction (WBS) algorithm. If high-frequency noise is removed in addition (steps 9 - 12), we denote the method wavelet-based background and noise subtraction (WBNS).

1. The input image (Python: tiff / Fiji: tiff, PNG, JPEG etc.) is loaded and converted to single precision (32 bit). Slices of an image stack are processed individually. Two parameters, the number of levels assigned as noise and the resolution parameter R are requested. R is given by the full width at half maximum of the PSF in pixel units, rounded to the next larger integer.
2. The image is converted to a new representation by decomposition into a set of coefficients using the discrete Haar wavelet transform. By applying a high-pass filter (defined by the Haar wavelet) and subsampling by a factor of two, the so-called "detail coefficients" are obtained, capturing the highest frequencies of the image. Application of a low-pass filter (quadrature mirror filter to high-pass) and subsampling yields the so-called "approximation coefficients" representing the low-frequency content. This procedure describes a single level of decomposition.
3. To extract and distinguish lower frequencies, the decomposition described in step 2 can be applied to the approximation coefficients of step 2. The approximation of level 1 is split into the approximation and details of level 2.
4. This procedure can be applied iteratively $\log_2(n)$ (rounded to the nearest smaller integer) times, where n is the width/height of the input image in pixels, resulting in a multiresolution representation of the input image. In practice, the process is stopped at a level associated with wavelet decomposition on the spatial scale of the signal content, which is roughly given by $\log_2(R)$, rounded to the next larger integer.
5. For (low-frequency) background extraction, the detail coefficients of the levels that contain high frequency information (characteristic length of the PSF or smaller) are set to zero.
6. The image is reconstructed from the modified wavelet coefficients by running the decomposition algorithm in reverse, which means applying the conjugate high-pass and low-pass filters to the approximation and detail coefficients.
7. The reconstructed image is filtered by a Gaussian filter with a standard deviation of 2^m, where m is the number of levels, smoothening discontinuities originating from the use of the (discontinuous) Haar wavelet.
8. The estimated background is subtracted from the input image; negative pixel amplitudes due to Gaussian filtering are set to zero.
9. The wavelet coefficients of the first decomposition level (step 2) are used to reconstruct an image containing only high frequency noise.
10. To this end, the approximation coefficients of the first level are set to one to create a flat background. The reconstructed image contains only high-frequency components, corresponding to spatial scales smaller than the width of PSF.
11. The noise image is corrected for outliers that may appear in bright regions of the image. Pixel values that exceed a threshold of 2σ (twice the standard deviation) are set equal to the threshold; negative numbers are set to zero.
12. The noise image is subtracted from the background-corrected image; negative numbers in the result are set to zero.
13. The pixel intensities are converted to the same precision as the input image before the image is saved.

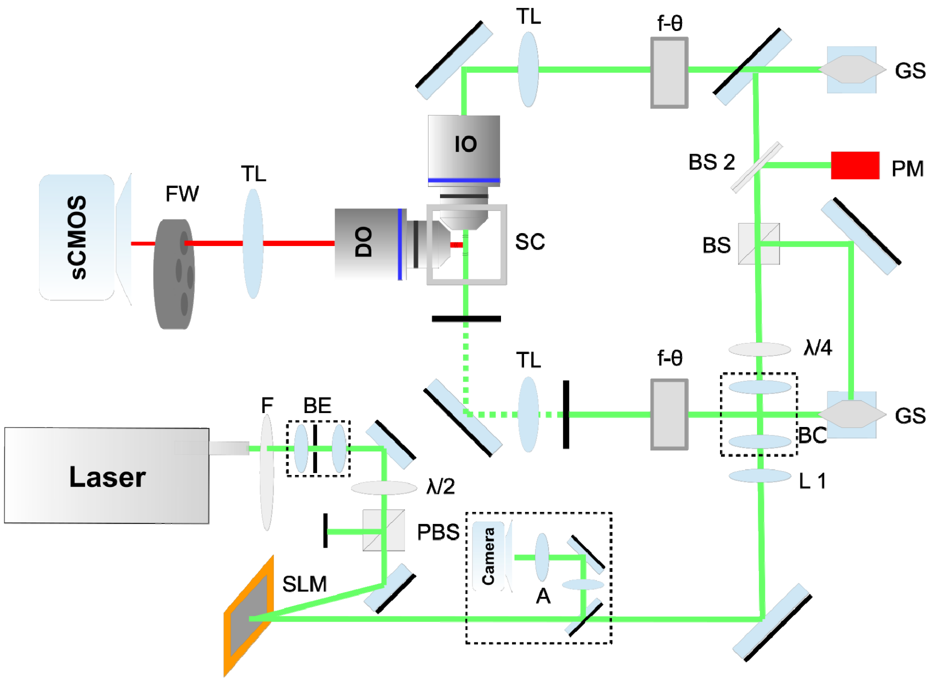

Fig. S2: Schematic of our digital light sheet microscope with confocal slit detection (DSLM-CS). The apparatus is an upgraded version of the device described in Ref. [1]. The key modification is the integration of a phase-only spatial light modulator (SLM) (LETO, Holoeye Photonics AG, Berlin, Germany) into the excitation beam path to enable flexible use of Gaussian or Bessel beams for light sheet preparation. Light from a 561 nm solid state laser (Jive, Cobolt, Hübner Photonics, Kassel, Germany) is filtered (F) (NDC-50C-2, Thorlabs GmbH, Dachau, Germany) and passed through a beam expander (BE) (AC254-010-A and AC254-080-A-ML, Thorlabs), so that a 10 mm diameter beam impinges on the SLM via a half-wave plate (λ/2, AHWP05M-600, Thorlabs) for power adjustment and a polarizing beam splitter (PBS) (PBS201, Polarized Beam Splitter, Thorlabs) to ensure the proper orientation of its polarization. Gaussian or Bessel beams are diffracted into the first order by loading a spherical (focal length f = 300 mm) or axicon (angle α = 0.5617°) lens phase pattern, respectively, on top of the diffraction grating pattern (spacing d = 32 µm) onto the SLM. An inspection camera setup (A) can be switched into the beam for diagnostic purposes. Two lenses (AC60 and AC100, Thorlabs) expand the beam and project it onto a CMOS camera (DCC1545M-GL, Thorlabs). The intermediate image plane located 300 mm from the SLM is projected onto the galvanometric scanning mirror (GS, GVS211/M, Thorlabs) and further to the back focal aperture of the illumination objective (IO, CFI PlanFluor 10×/0.3w, Nikon GmbH, Düsseldorf, Germany) via a spherical lens (L 1) with f = 500 mm (AC254-500-A-ML, Thorlabs), an f-theta lens (f-θ, S4LFT4375, f = 80 mm, Sill Optics, Wendelstein, Germany) and a tube lens (f = 200 mm, Nikon). In addition, there is a beam compressor (BC) (AC254-050-A-ML und AC254-040-A-ML, Thorlabs), a quarter-wave plate (λ/4) (AQWP05M-600, Thorlabs) to achieve circular polarization, a non-polarizing beam splitter (BS, 50:50, BS016,

Thorlabs) to generate two identical excitation pathways for two-sided illumination (not used in this work) inserted into the excitation path. A small amount of light is reflected onto a power meter (PM) (S121C, Thorlabs) by a second beam splitter (BS 2, 90:10) (BSF10-A, Thorlabs) for continuous measurement of the excitation power. The illumination objective is placed on a translation stage (CT1, Thorlabs) so that the region of minimal beam waist can be moved to a specific position within the sample. Both the illumination objective and the detection objective (DO, W plan Apochromat 63×/1.0w, Zeiss GmbH, Oberkochen, Germany) are firmly attached to the sample chamber (SC). The detection objective and the sample chamber are placed on a linear stage equipped with a motorized micrometer driver (M-462-X-SD, Newport GmbH, Darmstadt, Germany) for adjustment of the focal plane of the detection objective. There is also a sophisticated sample positioning system consisting of a motorized vertical translation stage (UZS80CC, Newport Darmstadt, Germany), a XY piezo stage (PILine M-686.D64, Physik Instrumente, Karlsruhe, Germany), and a rotor stage (SR2812s-20, SmarAct, Oldenburg, Germany). The emitted fluorescence is collected at 90° with respect to the plane of the light sheet, passed through a tube lens (TL, f = 200 mm, Nikon) and an emission band-pass filter (Semrock BrightLine HC 641/75, AHF, Tübingen, Germany) mounted in a filter wheel (FW, FW102C, Thorlabs) and is finally registered by a sCMOS camera (Orca Flash 4.0 v2, Hamamatsu Photonics GmbH, Herrsching, Germany). The camera is interfaced to a workstation consisting of a Supermicro X8DTG-QF mainboard, 2× Intel Xeon E5620 CPU, an Areca ARC-1880ix-12 SATA RAID controller and a multi-functional data acquisition card (NI PCI-6733, National Instruments, Austin, TX). All electronic components and real-time data acquisition are controlled by custom-written C++ based software. For confocal slit detection, galvanometric mirror movement and camera acquisition in progressive mode are synchronized.

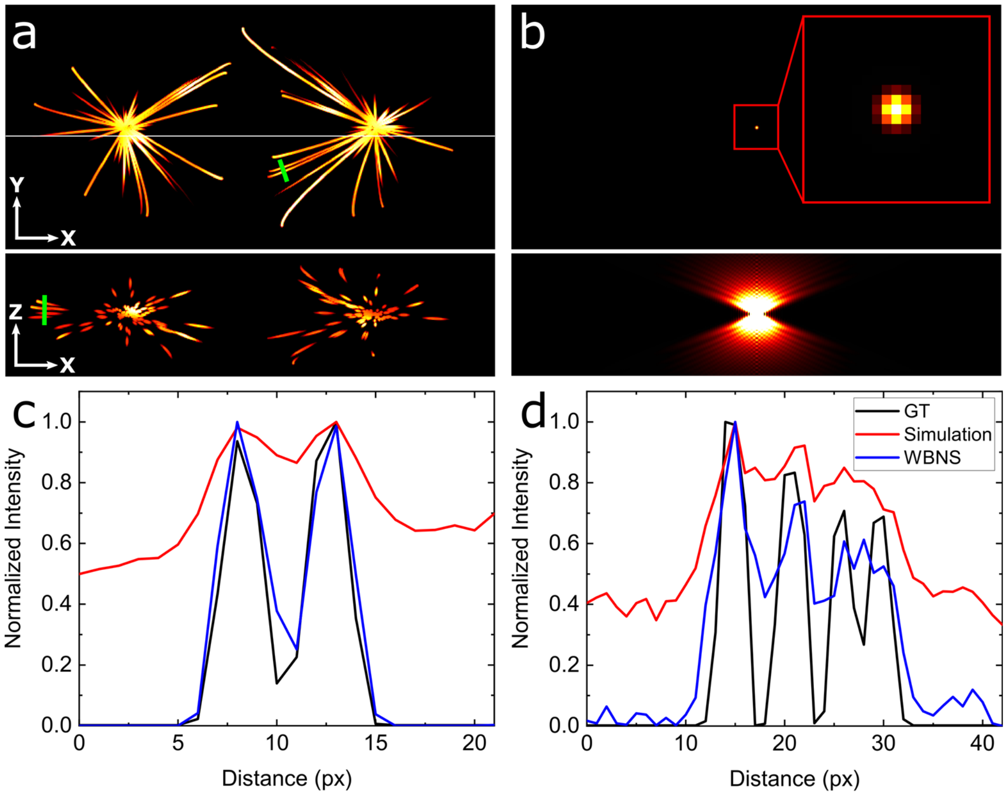

Fig. S3: Generation of synthetic images and WBNS application. (a) Slices through the ground-truth 3D data set; top: lateral (*x*,*y*) view; bottom: axial (*x*,*z*) view at the *y*-position marked in the top panel by the white horizontal line. (b) Lateral (top) and axial (bottom) views of the 3D widefield PSF used in the convolution with the ground-truth data set to calculate the simulated microscopy images in Figs. 2 and 3. The lateral extent of the PSF is ~3 pixels, so that a 2D convolution in the focal plane would only lead to small broadening. However, the 3D convolution generates substantial low-frequency background in the focal plane due to out-of-focus contributions. (c) Lateral and (d) axial views of line profiles along the green lines in panel (a), averaged over 5 pixels and peak-normalized to 1 for better comparison, showing data from the ground-truth (GT) image (black), the simulated microscopy image (red) and the image after WBNS processing of the synthetic image (blue). Note that WBNS effectively removes the low-frequency background, whereas the slight broadening of the sharp features due to the convolution (in the focal plane) of course persists.

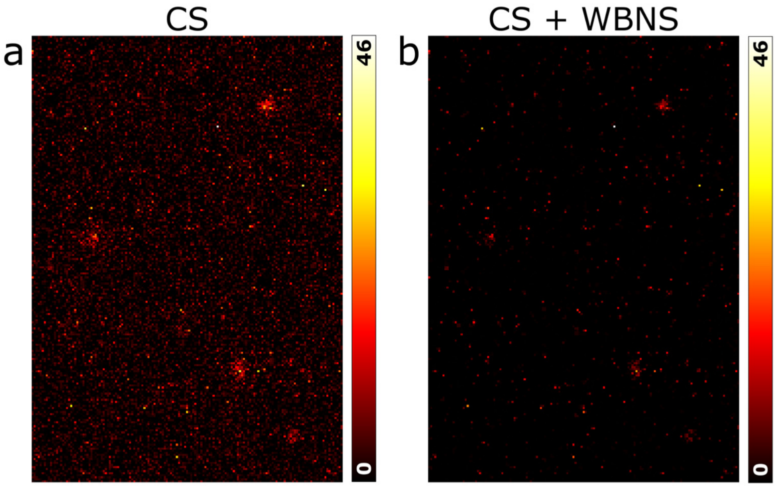

Fig. S4: WBNS on DSLM images taken with confocal slit detection. (a) Image shown in Fig. 4(b), reproduced here for ease of comparison with (b) the same image after processing with WBNS (R = 5 pixels), showing significantly reduced noise.

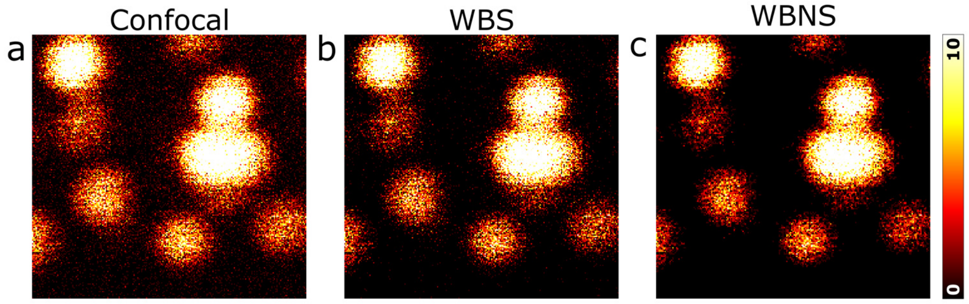

Fig. S5: Application of WBNS to a (heavily oversampled) confocal image. (a) Confocal image showing the same ROI as depicted in Fig. 5. (b) Image processed with WBS (R = 20 pixels). (c) Image after processing with WBNS (noise: first detail level). The contrast is enhanced to better visualize the high-frequency noise.

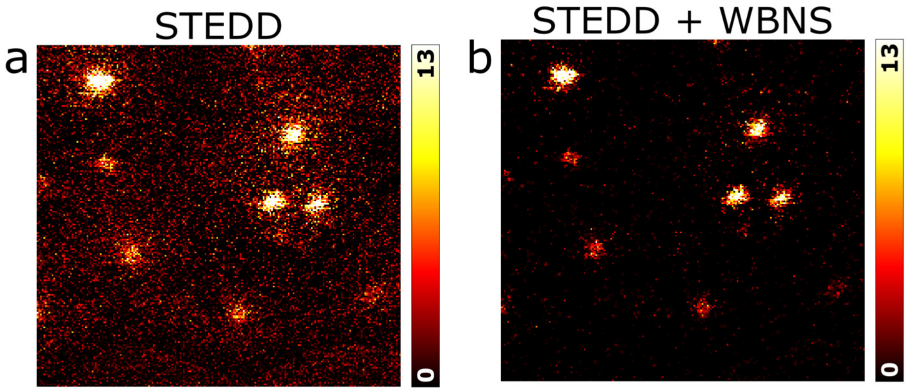

Fig. S6: WBNS on STED images with hardware-based background removal. (a) The STEDD image in Fig. 5(c) is reproduced here for ease of comparison with (b) the image after WBNS processing. In comparison to STED + WBNS, STEDD + WBNS yields additional noise and background suppression. Decorrelation analysis of this image returns a resolution of 95 nm and an A_0 value of 0.631 (*cmp.* Fig. 5(f)).

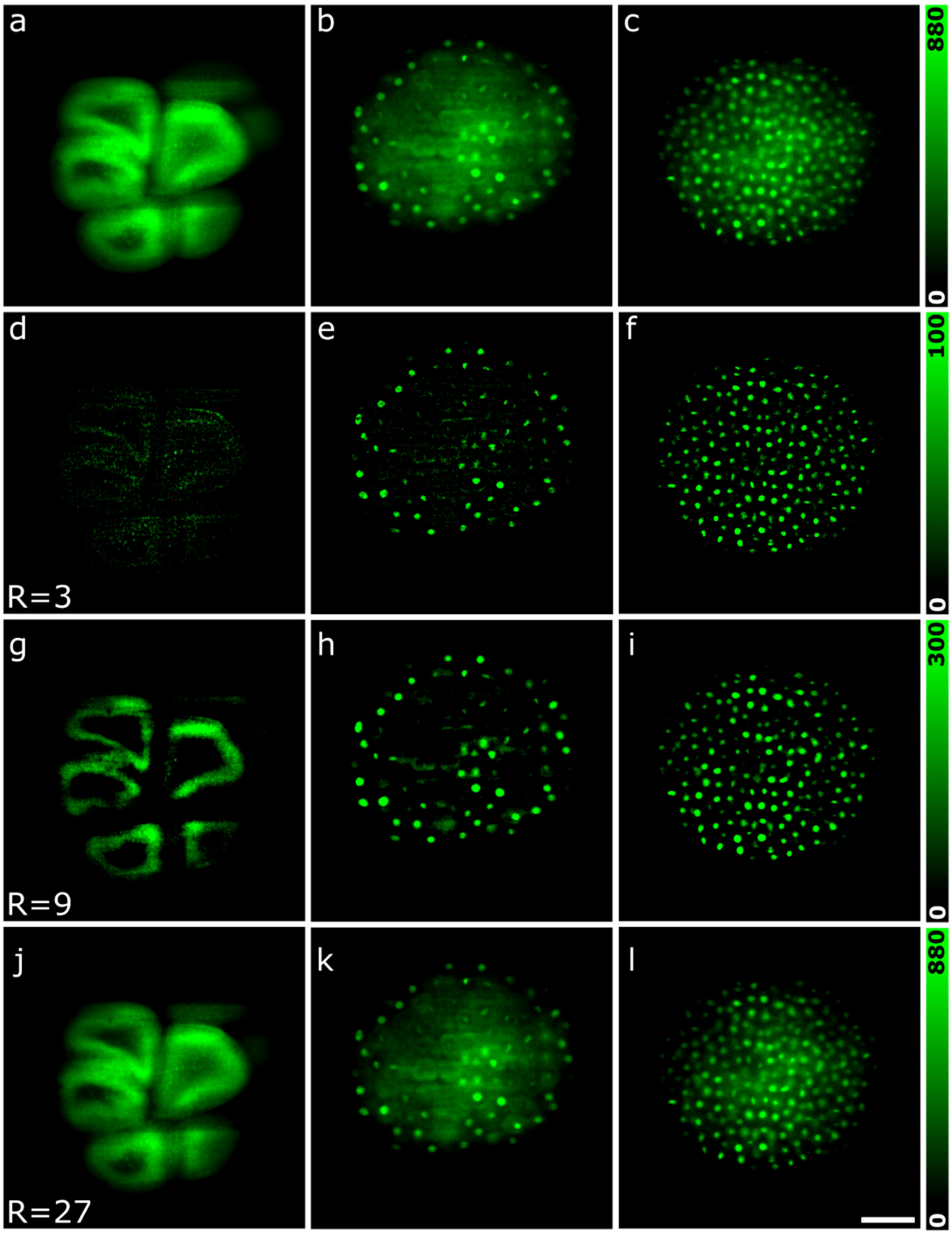

Fig. S7: The effect of using different *R* parameters for WBNS processing of DSLM images featuring image content widely dispersed in spatial frequency. Here we show slices of 3D image stacks of a developing transgenic zebrafish embryo expressing a histone 2a protein fused to the (enhanced) green fluorescent protein (EGFP) as the fluorescent label. At the earliest stages, the fusion protein appears in the cytoplasm. At the 256-cell stage, it starts translocating into the cell nucleus and at the 1024-cell stage, it mostly resides in the nucleus [2]. (a) At the 16-cell-stage, 1.5 hours post fertilization (hpf), the cytosol-stained cells generate low-frequency

image structures that are much larger than the PSF of the DSLM system. (b) At 2 hpf, cell nuclei appear as sharp bright spots, although cytosolic fluorescence is still visible. (c) At 2.9 hpf, protein translocation is complete, and the image shows stained nuclei plus background. (d, e, f) Images of the top row processed with WBNS using $R = 3$, corresponding to the image resolution. Panels (d) and (e) display a pronounced suppression of the low-frequency content of the image due to the small R parameter. By contrast, image background is correctly subtracted in panel (f). (g, h, i) Images of the top row processed with WBNS using $R = 9$. Consequently, the border between image and background is shifted to lower frequency, so that lower-frequency components are correctly assigned to image content and not removed as background. (j, k, l) Images of the top row processed with WBNS using $R = 27$. The even lower cut-off retains low-frequency image components in panels (j) and (k) but is no longer effective in background suppression in panel (l). Scale bar, 100 μm.

Supplemental Note 1: Software Guideline for the Python Implementation

System requirements:

The software was tested on workstations running under Microsoft Windows 7 and Windows 10. Only standard hardware is required. Adequate random access memory (RAM) is needed to process large data sets. Python version 3.7 was installed and we used the python distribution Anaconda 3 and Spyder 4 as python editor.

Installation:

The software is provided as a python script. Therefore, a python distribution (e.g. Anaconda 3) must be installed on the PC together with the following packages:

- Numpy
- Scikit-image
- Scipy
- Matplotlib
- pywavelets
- Joblib
- Multiprocessing
- tifffile

Using Anaconda, the packages can be installed by typing *conda install "package"* or *pip install "package"* into the IPython console. Installation of the packages may take up to 10 min.

Usage:

- Open WBNS.py in your python editor (e.g., Spyder 4)
- In the top section of the script, insert file location and file name of the image data (TIFF format).
- Input the resolution parameter *R* (in pixel units).
- Set the noise level parameter (number of levels used to extract noise). The default is "1". However, for low resolution images, "2" may yield better results.
- Run the script.
- Processed images will be saved in TIFF format in the same folder as the input data.

The resolution and noise level parameters are given in the "..._INFO.txt" file. Typical execution times for 3D demo data (Simulation_Microtubules_3D.tif) are 7 s on a computer with Intel(R) Core(TM) i7-8700K CPU; 32 GB RAM, and 21 s on a computer with Pentium(R) Dual-Core CPU E5700; 6 GB RAM.

Supplemental Note 2: Software Guideline for the ImageJ Implementation

System requirements:

The software was tested on workstations running under Microsoft Windows 7 and Windows 10. Only standard hardware is required. Adequate random access memory (RAM) is needed to process large data sets. ImageJ version 1.52v is required. We used Fiji as ImageJ distribution (http://fiji.sc/) [3, 4].

Installation:

Update ImageJ to version 1.52v if needed. In Fiji, click on "Help" → "Update ImageJ...", select "v.1.52v" and click "OK".

1. To run the macro, the Xlib (https://imagej.net/Xlib) library needs to be included to your update sites:
    a. In Fiji, click on "Help" → "Update.." → "Manage update sites".
    b. Activate the checkbox for "Xlib".
    c. Click "Close".
    d. Click "Apply changes".
    e. Restart Fiji.
2. Copy the "WBNS_ImageJ.ijm" file.
3. Paste it to "\fiji-win64\Fiji.app\plugins\" in your Fiji directory.
4. Restart Fiji.

Usage:

1) In Fiji, click on "Plugins" → "WBNS_ImageJ".

2) Follow the dialog.

Supplemental References

1. A. Y. Kobitski, J. C. Otte, M. Takamiya, B. Schäfer, J. Mertes, J. Stegmaier, S. Rastegar, F. Rindone, V. Hartmann, R. Stotzka, A. García, J. van Wezel, R. Mikut, U. Strähle, and G. U. Nienhaus, "An ensemble-averaged, cell density-based digital model of zebrafish embryo development derived from light-sheet microscopy data with single-cell resolution," Sci. Rep. **5**, 8601 (2015).
2. S. Pauls, B. Geldmacher-Voss, and J. A. Campos-Ortega, "A zebrafish histone variant H2A. F/Z and a transgenic H2A. F/Z: GFP fusion protein for in vivo studies of embryonic development," Dev. Genes Evol. **211**, 603-610 (2001).
3. J. Schindelin, I. Arganda-Carreras, E. Frise, V. Kaynig, M. Longair, T. Pietzsch, S. Preibisch, C. Rueden, S. Saalfeld, and B. Schmid, "Fiji: an open-source platform for biological-image analysis," Nat. Methods **9**, 676-682 (2012).
4. C. A. Schneider, W. S. Rasband, and K. W. Eliceiri, "NIH Image to ImageJ: 25 years of image analysis," Nat. Methods **9**, 671-675 (2012).
